# Differential tissue-specific protein markers of vaginal carcinoma

**DOI:** 10.1038/sj.bjc.6604975

**Published:** 2009-03-24

**Authors:** K Hellman, A A Alaiya, S Becker, M Lomnytska, K Schedvins, W Steinberg, A-C Hellström, S Andersson, U Hellman, G Auer

**Affiliations:** 1 Department of Gynaecologic Oncology, Radiumhemmet, Karolinska University Hospital, SE-171 76 Stockholm, Sweden; 2 Proteomics Unit, Stem Cell Therapy Program, King Faisal Specialist Hospital and Research Center, Box 3354, (MBC#03), 11211, Riyadh, Saudi Arabia; 3 Unit of Cancer Proteomics, Department of Oncology and Pathology, Karolinska Institute and Hospital, SE-171 76 Stockholm, Sweden; 4 Institute for Clinical Science, Department of Obstetrics and Gynaecology, Karolinska University Hospital, Huddinge, Karolinska Institute, SE-14186 Stockholm, Sweden; 5 Department of Gynaecology, Karolinska University Hospital, SE-171 76 Stockholm, Sweden; 6 Institution of Cytological Diagnosis (Kloster-Paradiese), Im Stiftsfeld, 159494 Soest, Germany; 7 Ludwig Institute for Cancer Research Ltd, Uppsala University, Box 595, SE-75124, Uppsala, Sweden

**Keywords:** vaginal carcinoma, cervical carcinoma, proteomics

## Abstract

The objective was to identify proteins differentially expressed in vaginal cancer to elucidate relevant cancer-related proteins. A total of 16 fresh-frozen tissue biopsies, consisting of 5 biopsies from normal vaginal epithelium, 6 from primary vaginal carcinomas and 5 from primary cervical carcinomas, were analysed using two-dimensional gel electrophoresis (2-DE) and MALDI-TOF mass spectrometry. Of the 43 proteins identified with significant alterations in protein expression between non-tumourous and tumourous tissue, 26 were upregulated and 17 were downregulated. Some were similarly altered in vaginal and cervical carcinoma, including cytoskeletal proteins, tumour suppressor proteins, oncoproteins implicated in apoptosis and proteins in the ubiquitin–proteasome pathway. Three proteins were uniquely altered in vaginal carcinoma (DDX48, erbB3-binding protein and biliverdin reductase) and five in cervical carcinoma (peroxiredoxin 2, annexin A2, sarcomeric tropomyosin kappa, human ribonuclease inhibitor and prolyl-4-hydrolase beta). The identified proteins imply involvement of multiple different cellular pathways in the carcinogenesis of vaginal carcinoma. Similar protein alterations were found between vaginal and cervical carcinoma suggesting common tumourigenesis. However, the expression level of some of these proteins markedly differs among the three tissue specimens indicating that they might be useful molecular markers.

Primary carcinoma of the vagina (PCV) accounts for approximately 1–2% of all gynaecological malignancies and predominantly affects postmenopausal women (Pettersson, 1999; Beller *et al*, 2006). However, the majority (85–90%) of malignancies arising in the vagina are metastatic lesions. Histologically most PCV cases are squamous cell carcinomas (Pettersson, 1999; Beller *et al*, 2006). As PCV is rare, little is known about aetiological and prognostic factors or about biological markers. The only established prognostic factor is tumour stage. It has been suggested that vaginal and cervical carcinomas share a common aetiology, because vaginal tumours tend to occur as a second primary malignancy in patients with a history of cervical dysplasia and/or neoplasia even after hysterectomy for these disorders (Choo and Anderson, 1982; Eddy *et al*, 1991; Kirkbride *et al*, 1995). Cervical squamous carcinomas are strongly associated with human papillomavirus (HPV) infection, with a prevalence of almost 100% (Bosch and Munoz, 2002). However, HPV-DNA has been identified in PCV in only about 50% of the cases (Daling and Sherman, 1992; Hildesheim *et al*, 1997). Cervical carcinomas occur in younger women, usually under the age of 60, whereas PCV is mainly found in older women. It has been proposed that PCV may develop in different ways, either through HPV-induced mutation or through other non-HPV factors that are also likely to play a role in the aetiology of PCV.

It is sometimes difficult to discriminate between PCV, cervical carcinoma and metastatic lesions, especially in patients with recurrent disease. Correct diagnosis is important for choice of therapy, prognosis and follow-up. Treatment and estimated tumour aggressiveness are still based on crude histopathological and clinical parameters, in both PCV and cervical carcinoma, resulting in individual cases of under- and over-treatment.

Increased biological knowledge of PCV and cervical carcinoma is needed to understand the molecular mechanisms of carcinogenesis and to find tumour-specific markers for diagnosis, prognosis and treatment.

Proteomic analysis provides an effective tool for understanding tumour biology and for establishing tumour-specific markers or protein profiles for certain clinical indications. Several genomic and proteomic studies have been carried out during the past decade to analyse gene expression and protein profiles for a variety of malignancies to find diagnostic, prognostic and predictive markers. 2-DE in combination with mass spectrometry has most commonly been used. Some tumour-specific markers for clinical use have been identified by proteomic studies, for example TAO1 and TAO2, are two effective markers for distinguishing primary lung adenocarcinoma from metastasised adenocarcinoma originating in other organs (Franzen *et al*, 1997; Hirano *et al*, 1997). These proteins were both later identified as napsin A (Chuman *et al*, 1999). Furthermore, cytokeratins and vimentin are used in the diagnosis of lung cancer and lower intestinal cancer (Cathro and Stoler, 2002; Liu *et al*, 2004).

Only a few proteomic studies have been conducted in cervical carcinoma (Bae *et al*, 2005; Choi *et al*, 2005; Lee *et al*, 2005; Lin *et al*, 2006), for which proteins such as annexin A2, tropomyosin 1 and 2, peroxiredoxin 2 and HSP 27 have been pointed out as potential diagnostic markers.

To date, only two studies have been carried out to investigate gene and protein expressions in vaginal carcinoma. The first study analysed the pattern of genomic imbalances in vaginal squamous cell carcinomas using comparative genomic hybridisation, which revealed that 70% of vaginal carcinomas carry relative copy number increases that map to chromosome arm 3q (Habermann *et al*, 2004). This pattern of genomic imbalances in PCV was strikingly similar to the one observed in cervical carcinomas (Heselmeyer *et al*, 1996) further strengthening the hypothesis of related aetiological pathways. Another study examined protein expression profiles using 2-DE in primary vaginal carcinoma compared with cervical carcinoma. Correlation analysis revealed high correlation between the tissue specimens. However, hierarchical cluster analysis correctly classified the samples in two groups (Hellman *et al*, 2004).

The purpose of this study was to identify the proteins that were differentially expressed in normal tissue and in vaginal and cervical cancer tissue by using the 2-DE protein separation technique and MALDI-TOF mass spectrometry to elucidate carcinogenetic pathways, to find out if they are functionally related and identify tumour-specific markers.

## Materials and methods

### Patient tissue samples, 2-DE gel electrophoresis and image analysis

Sixteen tissue biopsies, approximately 3 × 3 mm, were obtained and snap frozen. The samples consisted of five normal vaginal epithelium biopsies, as well as six primary vaginal carcinoma and five primary cervical carcinoma biopsies. This set of samples was previously described in a publication from our laboratory (Hellman *et al*, 2004). The histopathological characteristics of the samples are presented in Table 1.

The normal vaginal tissue samples were included as a baseline for the normal vaginal epithelium proteome and to ensure an effective comparison with vaginal cancer.

The rationale for including the cervical cancer samples was to elucidate similarities and differences between cervical and vaginal cancers at the proteome level.

All tumour biopsies were obtained from patients with histopathologically confirmed diagnosis of either vaginal or cervical cancers. To guarantee sample representativity, both cytological and histological evaluations were carried out on all samples, and only in those where both examined features corresponded were included in the study as previously described (Hellman *et al*, 2004). Informed consent was obtained before all sampling and the Stockholm County Council ethics committee gave its approval (Dnr: 97–244).

#### Sample preparation

All tissue samples were prepared according to the previously described method (Hellman *et al*, 2004). The proteins were extracted and solubilised in lysis buffer containing 9 M urea (BioRad, Hercules, CA, USA) 2 M thiourea (USB, Cleveland, OH, USA), 5% Resolyte (BDH, Poole, UK), 65 mM dithiothreitol (BioRad), 1 M EDTA (Merck, NJ, USA), 0.5% v/v NP-40 (Nonidet P-40; USB), 25 mM CHAPS, 0.1% PMSF (phenylmethylsulphonyl fluoride) and 1% benzamidine (Sigma, Missouri, USA). Protein concentration was determined by the Bradford protein analysis method (Bradford, 1976).

### Electrophoresis

#### First dimension

Iso-electric Focusing (IEF): The proteins are separated according to their iso-electric point in an immobilised pH gradient gel (IPG strip), see below.

#### Second dimension

Sodium dodecyl sulphate polyacrylamide gel electrophoresis (SDS–PAGE): The proteins that are focused in the immobilised pH gradient are further separated according to their molecular weight in a large size 10–13% gradient SDS–PAGE gel, 25 × 20 cm × 1.5 mm.

Before application, the first dimension samples were diluted to 0.25 mg ml^−1^ in rehydration buffer containing urea, thiourea, CHAPS, Triton X-100 (GE Healthcare, Amersham, UK) IPG buffer 4–7 (BioRad) and a few grains of bromphenol blue (Merck, ); 300 *μ*l were applied to 17-cm long IPG strips pH 4–7 (BioRad). IEF was performed in an IEF Cell (BioRad) for 22.5 h including rehydration until approximately 52 900 V h were reached.

The IPG strips were subsequently treated in two steps before loading the 2-DE gradient gels. The first step was to reduce the proteins using dithiothreithol and the second was equilibration to alkylate the free SH-groups using iodoacetamide (Sigma).

The second dimension, SDS–PAGE, was performed in gradient gels with piperazinediacrylamide (PDA; BioRad) as a cross-linker, in an ISO DALT tank (GE Healthcare) with the capacity to run 10 gels simultaneously. The electrophoresis was carried out at constant power, 100 V using an EPS2A200 power supply (Hoefer) at 12°C for approximately 19 h. After electrophoresis all gels were then incubated in fix solution, containing 30% ethanol (Kemetyl, Haninge, Sweden) and 10% acetic acid (Sigma), and subsequently stained with silver nitrate (Merck) (Rabilloud *et al*, 1994). Following development, a GS-710 (BioRad) flat bed densitometer was used to scan all high-resolution gels at a resolution of 105.8 *μ*m. The gels were matched to each other using PDQuest software, version 7.3.0 (BioRad). Background was subtracted and individual polypeptides quantified as p.p.m. of the total integrated optical density. Each spot was given a unique id number. All differentially expressed spots were individually examined to ensure that they were representative and were of high quality.

#### Excision

Proteins that were statistically significantly up- or downregulated by using Mann–Whitney statistical analysis and *t*-test (*P*<0.05), were selected for identification by mass spectrometry. Proteins were localised, excised manually, and transferred to sterile tubes. Some low abundant spots were pooled from different gels to increase the signal in mass spectrometric analysis. All excisions were performed in an LAF bench to minimise risk of contamination. Protective clothing, hair protection and extra arm protection were used. By maintaining these high standards keratins and other contaminants were kept low.

### Mass spectrometry

In-gel digestion for peptide mass fingerprint analysis was performed manually using trypsin (Hellman, 2002), and the digests were concentrated and desalted using *μ*ZipTip C18 (Millipore, Billerica, MA, USA) as recommended by the manufacturer. Peptides were eluted onto the MALDI target plate in 70% acetonitrile/0.1% trifluoroacetic acid containing half saturated *α*-cyano-4-hydroxycinnamic acid as matrix. Peptide mass fingerprinting was performed using MALDI-TOF MS on an Ultraflex TOF/TOF III (Bruker Daltonics, Bremen, Germany).

Trypsin autolytic fragments of charged masses 842.50, 2211.10 and 3337.76 Da were used as internal standards for spectra calibration. Data generated were screened in a protein sequence database (NCBInr, current version) using a mass tolerance ⩽20 p.p.m. Search engines used were Mascot (http://www.matrixscience.com) or ProFound (http://prowl.rockefeller.edu/prowl-cgi/). One missed cut was allowed and no restriction was made regarding species, size or pI; that is, the search was unbiased.

The above protocol for MALDI-TOF analysis has a sensitivity of low femtomole amounts of standard 2-DE gel-separated proteins. For a positive identification using peptide mass fingerprinting, protein scores greater than 72 were considered significant (*P*<0.05), as calculated by the MASCOT scoring algorithm. In addition, at least five matching peptides had to be found and more than 15% of the target protein's sequence had to be covered. We also considered and compared the practical and theoretical pI and Mw values of matching proteins.

### Data processing/data analysis

Both quantitative and qualitative 2-DE datasets were generated from PDQuest, a 2-DE software analysis program as previously described (Hellman *et al*, 2004).

The differentially expressed protein spots were selected for identification.

## Results

**Figure 1:**
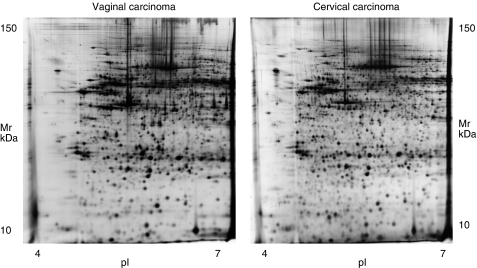
2-DE gel images of primary vaginal and cervical carcinoma.

Fresh-frozen tissue biopsies were subjected to 2-DE and analysed for both qualitative and quantitative differences in the expression of multiple proteins. An average total of 1373 spots were resolved on 25 × 20 cm 2-DE gels, and between 75 and 82% of the spots were matched among all gels. Silver stain was used to visualise gel spots (Figure 1).

Using PDQuest, the protein expression pattern in the different tissue specimens (normal vaginal tissue, vaginal cancer and cervical cancer) was analysed.

Mann–Whitney statistical analysis (*P*<0.05) revealed 67 proteins that were differentially expressed in normal vaginal tissue, vaginal cancer and cervical cancer. A similar analysis was carried out on these three groups of samples using Student's *t*-test, and 94 protein spots differed significantly. The differential analysis takes into consideration both the qualitative and quantitative changes observed between these sets of samples. Furthermore, these two separate datasets correctly classified the three groups of samples using hierarchical cluster analysis.

**Figure 2:**
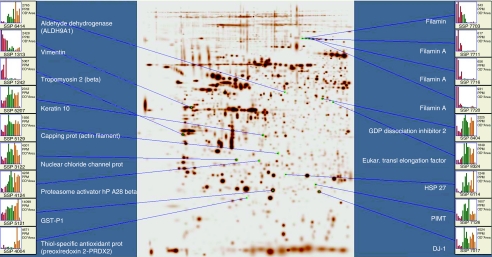
Global differential expression of some of the identified proteins between normal vaginal tissue, vaginal and cervical carcinoma (red=normal, green=vaginal cancer and orange=cervical cancer).

We excised a total of 104 spots with statistically significant variability in the expression pattern (*P*<0.05) between normal vaginal tissue, cervical cancer and vaginal cancer for identification by MALDI-TOF mass spectrometry. One obvious limitation when working with clinical samples is getting sufficient material for detailed analysis. Therefore, a relatively large number of the protein spots in the datasets for cluster analyses could not be identified. However, 61 proteins were clearly identified and among these, 43 proteins had prominent protein expression alterations (approximately 2- to 3-fold increase/decrease) as compared with normal tissue; 26 proteins were upregulated (Table 2) and 17 downregulated (Table 3). The differential expressions of some of these proteins are shown in Figure 2.

**Figure 3:**
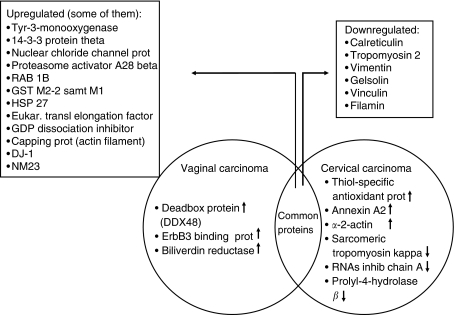
Venn diagram showing the proteins that were uniquely and similarly expressed in vaginal and cervical carcinomas. The arrows indicate up- or downregulation.

**Figure 4:**
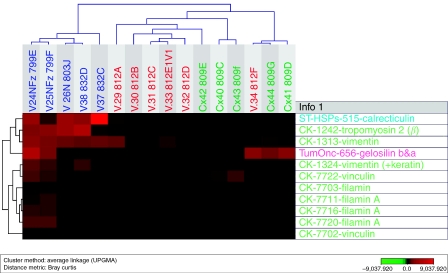
Cluster analysis using the expression patterns of 11 identified proteins that are downregulated in both vaginal and cervical carcinoma. Note that the majority are cytoskeletal proteins.

**Figure 5:**
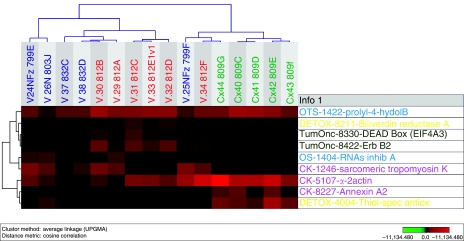
Cluster analysis using the expression patterns of nine identified proteins that are uniquely expressed in both vaginal and cervical carcinoma.

Of the 26 upregulated proteins, 3 were upregulated in vaginal carcinoma only, compared with normal vaginal tissue and cervical cancer (DEAD box protein (DDX48 or EIF4A3), erbB3-binding protein (Ebp1) and biliverdin reductase). In cervical carcinoma, three proteins were clearly upregulated compared with vaginal carcinoma and normal vaginal tissue (thiol-specific antioxidant prot, annexin A2 and alfa-2-actin). In both cancer specimens, 20 proteins were upregulated compared with normal vaginal tissue (tyr-3-monooxygenase, nuclear chloride channel prot, apolipoprotein (2), proteasome activator hP A28 beta, RAB 1B (RAS oncogen family), serpin B6 (thrombin inhib) (2), GST M2-2 as well as M1 (2), HSP 27, aldehyde dehydrogenase, (eukaryotic translation) elongation factor, GDP dissociation inhibitor 2, capping prot (actin filament), DJ-1, PIMT, chaperonin TCP1, 14-3-3 protein theta and NM23). Of the 17 downregulated proteins, 6 different proteins showed low expression in both vaginal and cervical carcinoma compared with normal vaginal tissue (calreticulin, tropomyosin 2 beta, vimentin, gelsolin, vinculin and filamin). Three proteins were distinctly downregulated in cervical carcinoma compared with vaginal cancer (sarcomeric tropomyosin kappa, Rnas inhib chain A and prolyl-4-hydrolase beta). For the distribution of proteins that were uniquely and similarly expressed in vaginal and cervical carcinoma see Figure 3. Furthermore, cluster analysis was performed using the expression patterns of some proteins that were downregulated (Figure 4) and uniquely expressed (Figure 5) in both vaginal and cervical carcinoma.

## Discussion

This study is the first proteomic study to analyse and identify proteins expressed in primary vaginal carcinoma and normal vaginal tissue by using MS. The expression profiles have also been compared with primary cervical carcinoma.

In all, we identified 43 proteins with significantly altered expression levels that discriminated well between non-tumourous and cancer tissue.

Thirty proteins showed similar trends of alterations in both vaginal and cervical cancer compared with normal vaginal tissue, which might indicate common carcinogenetic pathways and reflect the underlying pathogenesis to the diseases. However, three proteins were significantly altered in vaginal cancer exclusively (DEAD box, erbB3-binding protein and biliverdin reductase), and six proteins in cervical cancer (peroxiredoxin 2, annexin A2, sarcomeric tropomyosin kappa, Rnas inhib chain A and prolyl-4-hydrolase beta). These proteins may constitute valuable markers for diagnosis, prognosis or therapy, but this has to be confirmed in future studies.

Some of the proteins identified are discussed below and represent different categories involved in cellular pathways. Alterations were detected among tumour suppressor proteins, oncoproteins, proteins involved in the ubiquitin–proteasome pathway, in detoxification and antioxidation, stress-related proteins and cytoskeletal proteins. Some of these proteins have previously been described in other carcinomas, whereas others, to our knowledge, are not associated with these tumours.

### Tumour suppressor proteins and oncoproteins

#### The DEAD box protein

The DEAD box protein family of RNA helicases constitutes a large group of essential enzymes involved in all aspects of RNA metabolism, including transcription, splicing and translation (Abdelhaleem, 2004; Cordin *et al*, 2006). Based on their distribution patterns, members of this family are thought to be involved in different biological processes such as cellular growth and division (www.ncbi.nlm.nih.gov). Furthermore, these proteins may regulate p21^waf1/cip1^ transcription independently of p53 suggesting it may be a tumour suppressor gene (Chao *et al*, 2006).

The DEAD box protein 48 identified in vaginal carcinoma in this study has also been detected in pancreatic cancer and has been suggested as a potential serum marker (Xia *et al*, 2005).

#### ErbB3-binding protein 1

ErbB3-binding protein 1 (Ebp1) is a member of the family of proliferation-associated 2G4 proteins (PA2G4s) and plays a role in cellular growth and differentiation. It induces activation of the transmembrane receptor erbB3 (Kowalinski *et al*, 2007), which activates the ras pathway. The Ras–raf–mek pathway is the major downstream signalling pathway that emanates from all EGFR complexes. The EGFR signalling system comprises the four transmembrane proteins erbB1–erbB4, with intracellular tyrosine domains and extracellular ligand-binding domains. In vaginal cancer, the erbB3-binding protein was upregulated, indicating involvement of the ras pathway. In another study, Ebp1 overexpression resulted in downregulation of the androgen receptor leading to reduced incidence of androgen-dependent prostate tumours and slower tumour growth (Zhang *et al*, 2005b). To our knowledge this protein has not been described in any other carcinoma.

#### Ras 1B

Ras 1B was overexpressed in both vaginal and cervical cancer cells, which further points to involvement of ras oncogenes in the carcinogenesis of these malignancies.

The family of ras oncogenes has been extensively studied in human malignancies. Ras proteins play a direct causal role in human cancer and in other diseases. Mutations in the different ras oncogenes (H-Ras, N-Ras and K-Ras) occur in varying frequencies in different tumour types. RAS-mediated signals lead to effects on cell growth, differentiation, cycling and survival. Other cellular consequences of Ras activation include interactions with the Rho-family proteins. In gynaecological cancers, mutations of the K-Ras have been detected in endometrial (0–47%), cervical (0–61%) and ovarian carcinoma (0–46%; Mammas *et al*, 2005).

Alterations of other proteins that are supposed to function as tumour suppressors and oncoproteins were detected (14-3-3 proteins, RhoGDI2 protein, TEF1*δ*, DJ-1, Gelsolin and hnRNP H1).

#### The 14-3-3 proteins

The 14-3-3 proteins belong to an important, abundant highly conserved family that interact with many cellular proteins at their phosphorylation sites and the target proteins regulate various processes such as stress response, cell-cycle control, signal transduction and apoptosis (van Hemert *et al*, 2001; Hermeking, 2006). Seven different isoforms of these small acidic polypeptides of 28–33 kDa have been detected. One isoform 14-3-3 *σ* (=sigma) has been directly implicated in the aetiology of human cancer. It is induced by DNA damage and appears to be required for maintaining the G_2_/M checkpoint in epithelial cells, and its gene is directly regulated by p53 (Hermeking, 2003). Absent or decreased expression of 14-3-3 *σ* has been found in different cancers, such as vulvar cancer (Gasco *et al*, 2002), breast cancer (Urano *et al*, 2002), bladder cancer (Ostergaard *et al*, 1997) and neuroendocrine lung tumours (Yatabe *et al*, 2002).

In this study, three other isoforms were detected: 14-3-3 theta, zeta and epsilon, which might be implicated in the genesis of vaginal and cervical cancers. Previously, 14-3-3 epsilon and zeta proteins have been shown to play a role in RAS/MAP kinase pathways (Yip-Schneider *et al*, 2000). Similar to our findings, downregulation of the 14-3-3 epsilon protein has been described in cervical carcinoma (Bae *et al*, 2005), and overexpression of 14-3-3 zeta in lung cancer cells (Qi and Martinez, 2003) and oral squamous cell carcinoma (Matta *et al*, 2007).

#### GDP dissociation inhibitors

GDP Dissociation Inhibitors (RhoGDIs) are important regulators of the Rho proteins that sequester the GDP – bound Rho proteins in cytoplasm.

Rho proteins are involved in motility and metastasis of cancer cells through regulation of cell morphology and the actin cytoskeleton. RhoGDI2 has been identified as a metastasis suppressor gene, a marker of aggressive human cancer (Gildea *et al*, 2002). Genomic analyses of metastatic melanoma cells have shown overexpression of RhoC protein, indicating an important role of the protein in tumour invasion (Clark *et al*, 2000). Increased expression of Rho proteins has been detected in bladder cancer and seems to be involved in occurrence and progression of the cancer and may be valuable prognostic markers (Kamai *et al*, 2003).

In contrast to our results, reduced expression of RhoGDI2 has been demonstrated in bladder cancer (Theodorescu *et al*, 2004) and cervical SCC (Bae *et al*, 2005) and was associated with decreased survival in bladder cancer (Theodorescu *et al*, 2004) and lymph node metastasis in breast cancer (Hu *et al*, 2007).

The high expression of RhoGDI2 found in this study could possibly be explained by detection of these diseases at an early stage of cancer progression before downregulation of the GDP dissociation inhibitor 2 gene.

#### Eukaryotic translation elongation factor 1*δ*

Eukaryotic translation elongation factor 1*δ* (TEF1*δ*) is a newly discovered protein (Joseph *et al*, 2002) that has been shown to be a protooncogene causing tumourigenetic growth in nickel-treated human bronchial epithelial cells (Lei *et al*, 2006). High expression of TEF1*δ* was detected in NSCLC (Liu *et al*, 2004), which is consistent with our data.

#### DJ-1

is a 20 kDa ubiquitous cytoplasmic and nuclear oncogene product also known as PARK7. It is involved in multiple physiological processes including cancer, Parkinson's disease and male fertility (www.ncbi.nlm.nih.gov). According to a recent study overexpression of DJ-1 has protective effects on apoptosis associated with its ability to decrease the Bax level by inhibiting p53 transcriptional activity (Fan *et al*, 2008). High DJ-1 expression has been demonstrated in carcinomas of lung, breast and prostate (Hod, 2004; Fan *et al*, 2008), which is also consistent with our findings. In lung cancer this was correlated with poor clinical prognosis (Kim *et al*, 2005) and in breast cancer it has been associated with decreased expression of tumour suppressor phosphatase and deletion of the tensin homologue on chromosome 10 (PTEN; Kim *et al*, 2005).

#### Gelsolin

Gelsolin is an 82 kDa actin-binding protein. A gelsolin gene mutation has been found in familial amyloidosis (Haltia *et al*, 1992; Kamada *et al*, 1998). Studies have noted that overexpression of gelsolin prevent apoptosis (Ohtsu *et al*, 1997), whereas caspase-cleaved gelsolin can cause apoptotic cell death (Kothakota *et al*, 1997). Thus, gelsolin seems to have a dual function, as also proposed for Bcl-2 (Kamada *et al*, 1998). It has been noted that cells with overexpressed gelsolin have a prolonged S phase followed by a delay in G_2_, suggesting that downregulation of gelsolin may lead to malignant transformation by attenuating G_2_ checkpoint function (Sakai *et al*, 1999). Similar to our results, reduced expression of gelsolin has been detected in human gastric (TMK1) and urinary bladder (UMUC2) cancer cell lines, and has further been associated with mutated TP53 and shorter survival in bladder carcinoma (Sanchez-Carbayo *et al*, 2007).

### Antioxidation and detoxification

Several proteins involved in protein degradation, antioxidation and detoxification have been identified. Biliverdin reductase was upregulated in vaginal carcinoma, but not in cervical carcinoma. Upregulation of thiol-specific antioxidant was detected in cervical cancer, whereas prolyl-4-hydrolase beta was downregulated in cervical cancer.

Other antioxidants and detoxification proteins such as GST, Proteasome activator hP A28 beta, aldehyde dehydrogenase, serine protease inhibitor and PIMT have shown elevated expression in both vaginal and cervical cancer.

#### Biliverdin reductase

Biliverdin reductase (BVR) is a serine/threonine/tyrosine kinase that catalyses reduction of biliverdin to bilirubin. BVR is a zinc metalloprotein, thought to protect the cell from oxidative damage (Baranano *et al*, 2002; Lerner-Marmarosh *et al*, 2005). In concordance with our results, increases in biliverdin reductase expression have been detected in other carcinomas; renal cell (Maines *et al*, 1999) and hepatocellular carcinoma (Melle *et al*, 2007).

#### Thiol-specific antioxidants

Thiol-specific antioxidants (TSAs), also referred to as thiol peroxidases (TPx), comprise a novel family of thiol-specific antioxidant enzymes, that provides a protective role in cells via peroxidase (Shin *et al*, 2005). There are five peroxiredoxins (Prx) genes. TSA 1p was the first peroxiredoxin to be identified and has been shown to be the major thioredoxin peroxidase in cytoplasm (Wong *et al*, 2002). The members of the peroxiredoxin family have diverse functions associated with various biological processes including detoxification of oxidants, cell proliferation, cell differentiation and gene expression (Fujii and Ikeda, 2002; Jang *et al*, 2004). Altered expression of different peroxiredoxins has been identified in several malignancies, such as in hepatocellular carcinoma (Melle *et al*, 2007), cervical carcinoma (Bae *et al*, 2005), gastric carcinoma (Jang *et al*, 2004), clear cell ovarian adenocarcinoma (Morita *et al*, 2006) and prostate carcinoma (Ahram *et al*, 2002). In accordance with our findings, upregulation of TSAs (peroxiredoxin 2) has been detected in HPV16 E7-expressing cervical carcinoma cells (Lee *et al*, 2005). Studies have also shown that overexpression of peroxiredoxin 2 protein inhibits cisplatin-induced apoptosis, thereby contributing to chemoresistance in tumour cells (Chung *et al*, 2001).

#### Prolyl-4-hydrolase beta

Prolyl-4-hydrolase beta is a detoxification enzyme. Prolyl-4-hydrolase beta-isoform (P4HB) was found to be upregulated in HER-2/neu-positive breast tumours (Zhang *et al*, 2005a) in contrast to our findings.

#### The ubiquitin–proteasome pathway

The ubiquitin–proteasome pathway plays a major role in non-lysosomal degradation of dysfunctional intracellular proteins in eukaryotes. Proteins that are degraded by the ubiquitin–proteasome mechanism are first conjugated to ubiquitin. Ubiquitinated proteins are recognised by the 26S proteasome (Hasselgren and Fischer, 1997). Proteasomes can be activated by two different types of regulatory complexes. One is the proteasome activator hP A28 beta (Rivett *et al*, 1997). Alterations in ubiquitination and deubiquitination reactions have been directly implicated in the aetiology of many malignancies. Some of the natural substrates for degradation by the proteasome are oncoproteins that, if not properly removed from the cell, may induce malignant transformation (Ciechanover and Schwartz, 2004).

It has been noted that the tumour suppressor protein p53 level is extremely low in uterine cervical carcinoma tumours caused by high-risk HPV, (for example, HPVs 16 and 18). Studies have shown that the suppressor is targeted for ubiquitin-mediated degradation by the HPV oncoprotein E6 coded by high-risk strains of the virus (Scheffner and Whitaker, 2003). The viral oncoproteins exploit the ubiquitin–proteasome system to degrade and thus functionally inactivate negative cell-regulatory proteins including pRb and p53.

Upregulation of the 26S proteasome subunit has been detected in HPV16 E7expressing cervical carcinoma cells (Lee *et al*, 2005). In accordance with our results, upregulation of proteasome activator hP A28 beta have been detected in breast cancer (Somiari *et al*, 2003) and of proteasome activator complex subunit 2 in gastric cancer (Jang *et al*, 2004). Our results could support involvement of the ubiquitin–proteasome pathway in vaginal and cervical carcinogenesis.

#### Heat shock proteins

Heat shock proteins (HSPs) are required for cell survival under stressful conditions and are overexpressed in a number of human cancers. HSPs have antiapoptotic properties that protects the cell from programmed cell death and they are involved in cancer immunity (Calderwood *et al*, 2006; Multhoff, 2006). HSP 27 is principally found in cells from tissues such as breast, uterus, cervix, placenta and skin as well as in platelets (Ciocca *et al*, 1993). High expression in cancer cells has been shown to correlate with prognosis and drug resistance. Increased expression of HSP 27 has been identified in ovarian (Langdon *et al*, 1995; Geisler *et al*, 1999), cervical (Bae *et al*, 2005) and endometrial carcinoma and correlates with good prognosis. Similarly, in this study, HSP 27 was upregulated in the cancer specimens and this may also correlate with a good prognosis, because these cases were discovered at early stage. Conversely, HSP 27 overexpression has been recognised as a negative prognostic indicator in breast (Lemieux *et al*, 1997) and gastric (Ciocca *et al*, 1993; Kapranos *et al*, 2002) carcinoma.

#### Calreticulin

Calreticulin (CR) is a multifunctional calcium-binding protein present in the endoplasmic reticulum (ER) of the cell nucleus. It is an important molecular chaperone involved in ‘quality control’ within secretory pathways and has important regulatory functions such as modulation of steroid hormone receptors, retinoic acid receptors and regulation of cell adhesion. Increased levels of calreticulin have been reported in hepatocellular, colon, prostate and bladder carcinomas and as well as in radioresistant squamous carcinoma (Ramsamooj *et al*, 1995; Alaiya *et al*, 2000; Yoon *et al*, 2000; Brunagel *et al*, 2003; Iwaki *et al*, 2004).

In accordance with our results, downregulation of calreticulin has been noted in other squamous cell carcinomas, such as primary laryngeal and maxillar squamous cell carcinoma (Ogino *et al*, 2003, 2006).

Impaired p53 expression, function and nuclear localisation have been observed in calreticulin-deficient cells and the level of calreticulin (CRT) has been correlated with the rate of apoptosis (Mesaeli and Phillipson, 2004). Calreticulin plays a role in the control of cell adhesion via regulation of vinculin expression, which is downregulated in cells expressing reduced levels of calreticulin. Downregulation of calreticulin increases cell motility and decreases cell spreading (Opas *et al*, 1996).

### Cytoskeletal proteins

Alterations of several cytoskeletal proteins were identified in this study. Upregulation of annexin A2 was found in cervical cancer. In both vaginal and cervical carcinoma there was a downregulation of vimentin, filamin, tropomyosin 2 (TM2 beta) as well as vinculin.

The intermediate protein family includes several hundred members (such as cytokeratin, vimentin, actin, filamin, tropomyosin) and form the cytoskeletal network together with two other filament system (microfilament and microtubules). In high-grade dysplastic cervical cells filamin, vimentin and vinculin were upregulated (Gu *et al*, 2007) in contrast to the findings in cancer cells in our study.

#### Annexins

Annexins are calcium-dependent phospholipid-binding proteins and play a role in regulating the cytoskeleton and cell motility (Benz and Hofmann, 1997). Several annexins have been implicated in the pathogenesis of benign and malignant neoplasms of different origins. In some tumours a suppressive action of annexins has been shown, whereas in other tumours annexins have been involved in progression (Bastian, 1997; Hayes and Moss, 2004). Annexin A2 has been implicated in progression, migration and metastatic potential (Gillette *et al*, 2004; Hayes and Moss, 2004). Upregulation of annexin A2 (in line with our results) and A5 has been described in cervical carcinoma; however, annexin A1 was downregulated suggesting different roles for these annexins in the carcinogenesis of cervical squamous cell carcinoma (Bae *et al*, 2005). Upregulation of annexin A2 has been reported in many malignancies, such as buccal SCC (Chen *et al*, 2004), pancreatic, gastric, breast and brain cancer (Hayes and Moss, 2004; Tatenhorst *et al*, 2006). In renal carcinoma annexin 2 has been suggested as a useful prognostic marker (Zimmermann *et al*, 2004). However, reduced or lost expression of annexin 2 has been noticed in prostate carcinoma (Liu *et al*, 2003a) and osteosarcoma (Gillette *et al*, 2004) and has been related to aggressive behaviour and metastatic potential.

#### Tropomyosins

Tropomyosins (TMs) are actin-binding proteins. TMs isoforms and actin, are the two major structural constituents of microfilaments. The high-molecular-weight tropomyosins are thought to play a role in stabilising the organisation of actin filaments, which in turn plays an important role in the maintenance of cell shape, cell motility, and cell–cell and cell–matrix interactions (Button *et al*, 1995). Many TM isoforms, including tropomyosin-1 (TM1), are downregulated in transformed cells. The loss of tropomyosin expression in tumour cells may prevent proper assembly of microfilaments and, thereby contribute to the invasive and metastatic properties of cancer cells (Shah *et al*, 2001). In bladder carcinoma alterations in TM expression (reduced TM1, 2 and 3) seem to be an early event in bladder carcinogenesis (Pawlak *et al*, 2004). Similar to our findings, low expression of TM1 and TM2 has been detected in cervical carcinoma (Bae *et al*, 2005), of TM2 in oesophageal squamous cell carcinoma (Jazii *et al*, 2006) and of TM1 in breast carcinoma (Franzen *et al*, 1996; Raval *et al*, 2003). However, higher levels were found in primary breast cancer that had metastasised to the axillary lymph nodes (Franzen *et al*, 1996).

#### Alpha-2-actin

Alpha-2-actin, the human aortic smooth muscle actin gene, is one of six different actin isoforms, which have been identified. Actins are highly conserved proteins that are involved in cell motility, structure and integrity. Alpha actins are a major constituent of the contractile apparatus (www.ncbi.nlm.nih.gov). In this study, high expression of alpha-2-actin was noted in cervical carcinoma.

#### Vimentin

Vimentin is a type III intermediate filament protein that is frequently expressed in epithelial carcinomas and correlates with invasiveness and poor prognosis. Ablation of vimentin expression inhibits migration and invasion of colon and breast cancer cell lines (McInroy and Maatta, 2007) and promotes conversion of tumourigenic prostate epithelial cells into slow growing, less aggressive cells (Liu *et al*, 2003b). Upregulation of vimentin has been reported in prostate cancer, lung cancer and carcinosarcoma and vimentin have been used as a marker for lung cancer detection (Liu *et al*, 2004; Wu *et al*, 2007; Pardo *et al*, 2008; Wei *et al*, 2008). In colorectal tumours, increased vimentin expression correlates with the presence of oncogenic KRAS and with nuclear beta-catenin. However, corresponding liver, lymph node, brain and lung metastases did not express vimentin (Veenendaal *et al*, 2008), indicating other pathways in secondary colorectal tumours. Similar to the proteomic study of cervical carcinomas (Bae *et al*, 2005), but in contrast to the findings in other carcinomas, we found a downregulation of vimentin in both vaginal and cervical carcinoma, suggesting alternative pathways for epithelial–mesenchymal transition.

#### Vinculin and filamin

Vinculin and filamin are actin-binding proteins (ABPs) that provide structure and allow cells to be mobile. Studies have shown that filamin A integrates with beta-1 integrins to mediate cell spreading and prevent apoptosis (Kim *et al*, 2008). Furthermore 14-3-3 proteins bind both filamin and beta-2 integrin and induce distal signalling pathways leading to dramatic changes in the cytoskeleton, transcription and activation of integrins, which mediate adhesion (Nurmi *et al*, 2006). A previous study showed that filamin-A interacts with BRCA2, and lack of filamin-A expression results in increased cellular sensitivity to several DNA-damaging agents in melanoma cells (Yuan and Shen, 2001).

Vinculin is thought to function as one of several interacting proteins involved in anchoring F-actin to the membrane (www.ncbi.nlm.nih.gov).

Low levels of vinculin have been found in highly malignant neuroendocrine tumours. Therefore vinculin is thought to play a role in growth regulation of neuroendocrine tumours (Liu *et al*, 2007). Similarly, in this study, low levels of both vinculin and filamin were detected in vaginal and cervical carcinoma, indicating that these proteins are important for malignant transformation. There is not much data in the literature regarding vinculin and filamin expression in malignancies.

### Other proteins

#### Rnas inhib chain A

Rnas inhib chain A or human ribonuclease inhibitor (hRI) is an acid protein with a molecular weight of 50 kDa, which can inhibit the activity of pancreatic RNase (RNase A). Furthermore, RI is a highly efficient inhibitor of angiogenin, which is a member of the ribonuclease super family. Because angiogenin is an important angiogenic factor, it has been hypothesised that RI may be a latent antiangiogenic drug (Fu *et al*, 2005).

hRI was downregulated exclusively in cervical cancer. This finding may indicate that this protein is important for angiogenesis in cervical carcinoma and could possibly be an effective angiogenetic inhibitor in this specific type of cancer. To our knowledge this is the first time that this protein has been observed in cancer tissue.

#### The chloride intracellular channel

The chloride intracellular channel (CLIC) gene family has been implicated in chloride ion transport within various subcellular compartments (Berryman and Bretscher, 2000).

Antisense suppression of the chloride intracellular channel family induces apoptosis, and inhibits tumour growth (Suh *et al*, 2005). The cell- and tissue-specific patterns of CLIC1 expression suggest it may play distinct roles in different cell types (Ulmasov *et al*, 2007).

In accordance with our study elevated CLIC1 levels have been reported in hepatocellular carcinoma (Huang *et al*, 2004) and gastric carcinoma (Chen *et al*, 2007) and in gastric cancer this was strongly correlated with lymph node metastasis, lymphatic invasion, perineural invasion and pathological staging indicating that CLIC1 could be a potential prognostic marker (Chen *et al*, 2007). Otherwise, the literature contains little data on this protein and its relationship to cancer.

This study has shown alterations and interactions of several proteins, such as proteins involved in inactivation of p53 (DEAD box, the ubiquitin–proteasome pathway, 14-3-3 protein), activation of the RAS pathway (erbB3-binding protein) and proteins implicated in apoptosis (Dj-1, Gelsolin, hnRNPH1). Alterations of cytoskeletal proteins were another important finding not previously described for these carcinomas.

In accordance with our findings in cervical carcinoma Bae *et al*, 2005 detected upregulation of annexin A2 and downregulation of vimentin and tropomyosins 1 and 2, which might indicate that these cytoskeletal proteins are involved in tumourigenesis and could be useful markers. Interestingly, two previously relatively unknown proteins, the angiogenic factor human ribonuclease inhibitor and the detoxification enzyme prolyl-4-hydrolase beta, were distinctly downregulated in cervical carcinoma.

In summary, the protein variations identified in this study, indicate that numerous cellular pathways are activated leading to interaction of multiple proteins during vaginal and cervical tumourigenesis, which underscore the complexity of the cellular signalling networks responsible for the development and progression of cancer.

Some proteins were similarly altered in both vaginal and cervical carcinomas, suggesting common carcinogenetic pathways. However, certain proteins showed different levels of expression among the three tissue specimens, indicating that these could be specific tumour markers.

These data verify that proteomic analysis can provide extensive information to better understand the pathogenesis and oncogenesis of malignancies and further provide a basis to develop useful molecular markers for diagnosis, prognosis and therapy. The fact that multiple cellular pathways seem to be involved indicates that multiple proteins should be simultaneously targeted. However, further studies are needed to confirm the results from this study.

## Figures and Tables

**Table 1:** Clinical and histopathological data

**No**	**Sample**	**Type of sample**	**Age**	**Stage**	**Histology**	**Grade**
1	V24N	Normal vaginal tissue	48			
2	V25N	Normal vaginal tissue	70			
3	V26N	Normal vaginal tissue	56			
4	V37N	Normal vaginal tissue	78			
5	V38N	Normal vaginal tissue	78			
6	V29T	Vaginal carcinoma	83	I	SCC	
7	V30T	Vaginal carcinoma	54	I	AC	
8	V31T	Vaginal carcinoma	89	I	SCC	
9	V32T	Vaginal carcinoma	62	I	SCC	Low
10	V33T	Vaginal carcinoma	78	I	SCC	Medium
11	V34T	Vaginal carcinoma	75	IIB	SCC	High
12	Cx40	Cervical carcinoma	45	IIB	SCC	Low
13	Cx41	Cervical carcinoma	41	1B2	AC	
14	Cx42	Cervical carcinoma	63	IIA	SCC	Low
15	Cx43	Cervical carcinoma	59	IB2	SCC	Medium
16	Cx44	Cervical carcinoma	65	IB1	SCC	Medium

SCC=squamous cell carcinoma; AC=adenocarcinoma.

**Table 2:** Upregulated proteins (26) in vaginal and cervical carcinoma compared to normal vaginal tissue

		**Arbitrary (relative) intensity^a^**			**Empiric**		**In silica**				
**Sample ID**	**Result**	**Normal**	**Vaginal cancer**	**Cervical cancer**	**pI**	**MW**	**pI**	**MW**	**Accession number**	**% Sequence coverage**	**Number of matched peptides**
1107	14-3-3 protein theta	2	4	3	4.5	30	4.7	28	NP_006817	45	17
1123	Tyr-3-monooxygenase (14-3-3 zeta)	2	4	3	4.8	30	4.8	28	NP_036611	47	11
3122	Nuclear chloride channel prot	2	4	4	5.3	32	5.0	27	AAD26137	43	7
4004	Thiol-specific antioxidant prot (peroxiredoxin 2)	1	1	4	5.6	20	5.9	18	CAA57764	24	5
4103	Proapolipoprotein	2	4	4	5.5	25	5.4	29	AAA51747	76	29
4124	Proteasome activator hP A28 beta	2	4	4			5.4	28	AAF02218	37	10
4134	RAB 1B (RAS oncogen family)	1	4	4	4.6	22	5.6	22	NP_112243	46	8
4306	Serpin B6 (thrombin inhib)	2	3	3	5.6	60	5.2	43	NP_109591	27	9
5107	Alfa-2-actin	2	2	4	5.9	30	5.2	42	NP_001604	41	13
5114	Apolipoprotein A1	2	3	3	5.8	30	5.6	31	NP_000030	27	7
5121	GST (GSTP1_HUMAN)	2	4	4	5.9	20	5.5	23	CAA30894	61	13
5129	Capping prot (actin filament) (CAPZB)	2	4	4	6	30	6.5	30	CAH71390	31	9
6016	NM23 (nucleoside difosfatkinase)	2	4	3	6.2	15	5.8	20	NP_937818	32	5
6114	HSP 27	2	4	4	6.1	30	6.0	23	NP_001531	41	9
6414	Aldehyd dehydrogenase (ALDH9A1)	2	4	3	6.1	80	5.4	52	AAB06721	15	8
7017	DJ-1	2	4	4	6.6	20	6.3	20	NP_009193	56	11
7126	PIMT	2	4	3	6.4	20	6	25	AAA90934	44	7
7312	Serine peptidase inhibitor (Serpin B6)	2	3	2	6.2	65	5.9	43	NP_109591	42	14
8114	GST M2-2 samt M1	2	4	3			6.0	26	Gi4557966	71	16
8211	Biliverdin reductase A	2	4	2	6.6	55	6.1	34	AAH05902	20	6
8227	Annexin A2	1	1	4	6.9	50	7.7	39	AAH09564	18	6
8324	Eukar. Transl Elongation factor	2	4	4	6.7	80	6.3	50	NP_001395	33	13
8330	DEAD box (eukaryotic translation initiation factor 4A, isoform 3 (EIF4A3), eller DDX48 mRNA).	2	4	2	6.7	80	6.3	47	NP_055555	23	12
8404	GDP dissociation inhibitor 2	2	4	4	6.7	80	5.9	46	CAI13363	49	16
8422	Erb B2 binding protein (erbB3 binding protein EBP1)	2	4	2	6.7	80	7.2	38	AAD00646	18	12
8511	Chaperonin (containing TCP1, subunit 3 (gamma))	2	3	3	6.6	85	6.1	61	AAH08019	34	18

1, extremely low; 2, low; 3, moderately; 4, high;

a Arbitrary (relative) intensity.

**Table 3:** Downregulated (17) proteins in vaginal and cervical carcinoma compared to normal vaginal tissue

		**Arbitrary (relative) intensity^a^**			**Empiric**		**In silica**				
**Sample ID**	**Result**	**Normal**	**Vaginal cancer**	**Cervical cancer**	**pI**	**MW**	**pI**	**MW**	**Accession number**	**%sequence coverage**	**Number of matched peptides**
515	Calreticulin	4	2	2	4.4	95	4.3	48	NP_004334	63	20
1105	14-3-3 protein epsilon	4	4	3	4.6	30	4.6	29	NP_006752	57	16
1242	Tropomyosin 2 (beta)	4	1	1	4.7	45	4.6	33	NP_998839	58	24
1246	Sarcomeric tropomyosin kappa	4	4	1	4.6	60	4.7	33	AAT68294	50	22
1313	Vimentin	4	1	1	4.8	65	5.1	54	CAA79613	36	20
1324	Vimentin (+keratin)	4	1	1	5	60	5.1	53	CAA79613	53	31
1404	Rnas inhib chain A	4	4	1	4.6	80	4.7	52	NP_002930	65	21
1422	Prolyl-4-hydrolase beta	4	4	1	4.9	80	4.8	58	NP_000909	45	22
6439	hnRNP H1	3	4	2	6.2	80	5.8	49	CAG33059	37	12
6615	Gelsolin b och a	4	2	2	6	100	5.6	83	NP_937895	36	24
7702	Vinculin	4	2	2 (3)	6.7	60	5.6	88	CAI39669	14	9
7703	Filamin	4	2	1	6.2	45	5.9	89	AAH14654	22	12
7711	Filamin A	4	1	1	6.2	>100	5.7	105	AAH67111	34	23
7716	Filamin A	3	1	1	6.3	>100	5.7	280	BAD52436	14	24
7720	Filamin A	3	1	1	6.3	>100					
7722	Vinculin	4	2	3	6.4	100	5.8	117	NP_003364	38	34
9511	WD repeat	3	3	2	6.9	95	6.2	67	NP_059830	58	33

1, extremely low; 2, low; 3, moderately; 4, high;

a Arbitrary (relative) intensity.
